# TLR/NCR/KIR: Which One to Use and When?

**DOI:** 10.3389/fimmu.2014.00105

**Published:** 2014-03-19

**Authors:** Simona Sivori, Simona Carlomagno, Silvia Pesce, Alessandro Moretta, Massimo Vitale, Emanuela Marcenaro

**Affiliations:** ^1^Dipartimento di Medicina Sperimentale, Centro di Eccellenza per le Ricerche Biomediche, Università degli Studi di Genova, Genova, Italy; ^2^IRCCS Azienda Ospedaliera Universitaria San Martino-IST, Istituto Nazionale per la Ricerca sul Cancro, Genova, Italy

**Keywords:** NK cell, TLR, KIR, NCR, CCR7, anti-tumor response, anti-viral response, innate immunity

## Abstract

By means of a complex receptor array, Natural killer (NK) cells can recognize variable patterns of ligands and regulate or amplify accordingly their effector functions. Such NK receptors include old, rather conserved, molecules, such as toll-like receptors (TLRs), which enable NK cells to respond both to viral and bacterial products, and newer and evolving molecules, such as killer Ig-like receptors and natural cytotoxicity receptors, which control NK cytotoxicity and are responsible for the elimination of virus-infected or tumor cells without damaging self-unaltered cells. In addition, to rapidly gain new functions NK cells also can acquire new receptors by trogocytosis. Thus, NK cells may have adapted their receptors to different functional needs making them able to play a key role in the modulation of critical events occurring in several compartments of human body (primarily in SLCs but also in decidua during pregnancy). In this review, we will discuss on how the various types of receptors can be used to address specific functions in different immunological contexts.

## Introduction

Natural killer (NK) cells are innate lymphocytes present in all mammalian species capable of mediating multiple effector functions. NK cells express a number of receptors through which they can directly recognize microbial products, can sense aberrant/transformed cells [which lack constitutive self-human leukocyte antigen (HLA)-I molecules and express ligands for activating NK receptors], or even mediate regulatory functions (being an early source of cytokines) (1, 2).

Despite the original definition of NK cells based on their “natural” cytotoxicity against transformed cells in the absence of prior immunization, several studies indicate that NK cells require education/maturation process before killing and carrying out their biological functions (3).

Specialized NK subsets, which display distinct functions according to their typical cell-surface phenotype, exist. In particular, the CD56^bright^ NK cell subset presents little cytolytic activity but releases high amounts of cytokines, whereas CD56^dim^ NK cell subset displays potent cytotoxicity but also high cytokine production in response to specific stimuli (4–6).

The former subset is characterized by the CD16^−^KIR^−^NKG2A^+^ phenotype and largely predominates in lymph nodes, according to the expression of CCR7 (the homing receptor for secondary lymphoid compartments, SLCs) (7, 8), whereas the latter subset is CD16^+^KIR^+^ and/or NKG2A^+^ and prevails in peripheral blood and inflamed tissues where they can be recruited, thanks to the expression of the CXCR1, CX3CR1, and ChemR23 chemokine receptors (4, 9, 10).

Thus, during the early phases of an inflammatory response, CD56^dim^ NK cells may be recruited into inflamed tissues in response to various chemokines. Notably, the extravasation of NK cells do not imply their activation, thus these cells, once reached inflammatory sites, need to be activated to carry out their full effector functions (9). Candidates for NK cell activation are cytokines, released by innate cells, which are known to interact with NK cells (e.g., IL12), and/or engagement of certain activating receptors, including toll-like receptors (TLRs) and/or natural cytotoxicity receptors (NCRs) (11–14).

The stimulatory activity mediated by these receptors can induce NK cell-mediated lysis of tumor/virus-infected cells, the release of pro-inflammatory cytokines and, importantly, can promote the interactions of NK cells with other innate cells (15–17). This latter event can help to boost the innate immune system and promote the development of efficient adaptive immune responses (18).

In this review, the contribution of the different types of NK cell receptors in NK cell activation and their cooperation has been analyzed. In addition, new mechanisms of cell communication that allow the acquisition of unexpected receptor functions and/or novel functional properties have been described (7, 19, 20).

These novel aspects, probably occurring in response to environmental stress such as viral or bacterial infections, disclose new potential implications of NK cells in physiologic and pathologic conditions.

## Toll-Like Receptors

Toll-like receptors belong to pattern-recognition receptors (PRRs), which have evolved to recognize conserved features of microbes, the so-called pathogen-associated molecular patterns (PAMPs) (21, 22). TLRs are expressed on NK cells independently of their state of activation and can synergize with chemokine- or cytokine-mediated signals to activate NK cell function (Table 1A). Both NK cells and other innate cells express certain TLRs, thus allowing a coordinated response to the same pathogen-derived product. For example, viral dsRNA can act on TLR3 expressed by both myeloid dendritic cells (DCs) and NK cells, recruited by chemokine gradients to inflammatory sites. In the presence of IL12 (released by DCs after TLR3 stimulation), NK cells respond to dsRNA by improving their killing capabilities: not only against abnormal target cells, but also against immature DCs (iDCs) (13). This latter effect has been proposed as a mechanism by which NK cells can “edit” the process of DC maturation by selecting those DCs that are undergoing appropriate maturation and therefore would best prime T cells after migration to SLCs (23, 24). Moreover, upon the simultaneous stimulation by TLR3 engagement and IL12, NK cells also increase their capability of secreting pro-inflammatory cytokines, which mediate several important functions, including promotion of further DC maturation, anti-viral/anti-tumor effects, and induction of Th1 responses (13, 15, 18).

**Table 1:** **(A) TLRs expressed by human NK cells and relative ligands: effect of their interaction; (B) NCRs expressed by human NK cells and relative ligands: effect of their interaction; (C) KIRs expressed by human NK cells and relative ligands: effect of their interaction**.

	Ligand(s) type	Ligand(s) expression	Effect of receptor/ligand interaction
**(A) TLR**			
TLR2	Bacterial lipoprotein (BCG)	Bacteria (27, 28)	Induction of cytotoxicity and cytokine release
TLR3	Double-stranded RNA	Viruses (13)	Induction of cytotoxicity and cytokine release
TLR5	Flagellin	Bacteria (33)	Induction of cytotoxicity and cytokine release
TLR7/8	Single-stranded RNA	Viruses (34, 35)	Induction of cytotoxicity and cytokine release
TLR9	CpG DNA motifs	Bacteria/viruses (13)	Induction of cytotoxicity and cytokine release
**(B) NCR**			
NKp30	B7-H6	Tumor cells (40)	Activation (cytokine/cytotoxicity)
		Monocytes/neutrophils (48) (induced by TLR engagement)	Activation/regulation?
	BAT3/BAG6	Tumor cells (39)	Activation (cytokine/cytotoxicity)
		DC (47) (induced by TLR engagement)	Activation of NK/DC cross-talk
	ECTV HA	Infected cells (44)	N.D.
	VV HA	Infected cells (44)	Inhibition
NKp44	21spe-MLL5	Tumor cells (41)	Activation (cytokine/cytotoxicity)
	Influenza virus-HA; Sendai virus-HN	Infected cells (42)	Activation (cytokine/cytotoxicity)
	PCNA	Induced on tumor cells (114)	Inhibition
	N.D.	N.D.	IL-22 and TNFα release by ILC3
	N.D.	BCG (29)	Supporting of NK cell activation
	N.D.	Trophoblasts (73, 74)	Activation (cytotoxicity)
NKp46	N.D.	Tumor cells (14, 37)	Activation (cytokine/cytotoxicity)
	Influenza virus-HA; Sendai virus-HN	Infected cells (38, 44)	Activation (cytokine/cytotoxicity)
	N.D.	CMV-infected DC (51)	Activation (cytokine/cytotoxicity)
	NDV-HN	Infected tumor cells (115)	Activation (cytokine/cytotoxicity)
	N.D.	Trophoblasts (73, 74)	Chemokine production by dNK
**(C) KIR**			
2DL1	C2 epitope	Normal cells	Inhibition
2DL2/DL3	C1/C2 epitope; few HLA-B (C1 epitope)	Normal cells	Inhibition
2DL4	HLA-G	Normal cells	Inhibition/IFNγ induction
	CpG ODN (+ +)	Infected cells?	N.D.
2DL5	N.D.	N.D.	Inhibition
2DS1	C2 epitope	Normal cells; Tumor cells	Activation (cytokine/cytotoxicity)
2DS2	N.D.	N.D.	N.D.
2DS3	N.D.	N.D.	N.D.
2DS4	N.D.	Melanoma cell lines; primary melanoma	Activation (cytokine/cytotoxicity)
	HLA-A11; some C1 and C2 HLA-C	N.D.	Activation (cytokine/cytotoxicity)
2DS5	N.D.	N.D.	N.D.
3DL1	HLA-B and HLA-A (Bw4+)	Normal cells	Inhibition
	CpG ODN (+ + +)	Infected cells?	N.D.
3DL2	HLA-A3, −A11	Normal cells	Inhibition
	CpG ODN (+ + +)	Infected cells?	Activation (cytokine/cytotoxicity)
3DL3	N.D.	N.D.	N.D.
3DS1	CpG ODN (+ + +)	Infected cells?	N.D.
	HIV-peptides?; HCV-peptides?	Infected cells?	N.D.

*ECTV, ecteromelia virus; VV, vaccinia virus; NDV, new castle disease virus; dNK, decidual NK cells; N.D., not determined*.

Similar to DCs, NK cells also express TLR2, specific for products of bacterial origin (25). TLR2 is directly involved in the NK-mediated recognition of *Mycobacterium tuberculosis* (BCG) by NK cells (26–28). In turn, DCs, when exposed to BCG, release large amounts of IL12 that induce an amplification of the effector functions of NK cells. These include the enhancement of the NK cell cytotoxicity against both tumor cells and iDCs, and the cytokine release that can promote maturation of DCs, capable of inducing adaptive Th1 immune responses (27).

Moreover, the TLR2-mediated interaction of NK cells with BCG may induce the NK cell expression of NKp44, which, in turn, can directly bind to BCG (29). However, while TLR2 binding to *Mycobacterium* cell wall is sufficient to induce activation of NK cell effector functions (including IFN-γ production), the engagement of NKp44 by BCG cell wall components may play a role in maintaining NK cell activation (28).

In addition, it has been recently demonstrated that TLR2 may be also involved in the NK-mediated response to human CMV (30).

Microbial unmethylated CpG DNA motifs are able to stimulate both NK cells and plasmacytoid DCs (pDCs) via TLR9, which is, indeed, expressed by both cell types. IFN-α, released by pDCs upon TLR9 engagement, supports the triggering of TLR9-responsive NK cells (31, 32). This activation may be further amplified by IL12, released by DCs (31).

It has been reported that NK cells may also express TLR5. Flagellin, a typical TLR5 ligand, may directly act on NK cells, and induces the release of IFN-γ, contributing to activate surrounding cells, and α-defensins, mediating pathogen destruction (33).

Human NK cells may also express functional TLR7 and TLR8 (34). In this context, it has been shown that NK cell stimulation by the TLR7/8 ligand ssRNA derived from HIV-1 depends on a direct contact between NK cells and pDCs or monocytes (35).

Thus, although NK cells can be directly activated by some TLR agonists, the microenvironment in which they lie, during TLR-mediated activation, may play an important role not only in the activation of their cytotoxic activity but also in their regulatory functions, able to modulate subsequent adaptive immune responses (11, 22, 36).

## Natural Cytotoxicity Receptors

NKp46, NKp44, and NKp30 were among the first identified activating receptors on human NK cells. These structurally unrelated surface molecules were collectively defined as NCRs for their common ability to strongly activate NK cell cytolytic activity (37). The generation of NCR-specific blocking monoclonal antibodies (mAbs) and the identification of an NCR^dim^-phenotype (with impaired NK-mediated tumor killing capabilities) in some individuals (14), rapidly allowed the demonstration that these receptors were recognizing ligands on a large array of NK-susceptible targets, primarily tumor cells. The first NCR ligands to be discovered, however, were of viral origin (38), while only recently some of the tumor-expressed cellular ligands (39–41) have been identified (Table 1B). Different viral hemagglutinins (HAs) bind one or more NCRs and trigger NK cell functions (42). The pressure exerted by NCRs on viruses is witnessed by the onset of specific escape mechanisms (43, 44). Thus, for example, the CMV-encoded pp65 molecule gives rise to intracellular inhibitory interaction with NKp30 (43) and the vaccinia virus HA has been recently shown to bind NKp30 and block NKp30-mediated activation (44). In addition, NK cells in HIV-infected patients may show various alterations, including a reduced expression and function of NCRs (45, 46).

The so far identified tumor-expressed NCR-ligands are represented by self-antigens whose expression/exposure at the outer cell surface can be induced by cell stress or activation, or by still unknown mechanisms related to tumor transformation (Table 1B). Thus, the NKp30-ligand HLA-B-associated transcript-3/BCL-2-associated athanogene 6 (BAT3/BAG6) is a nuclear factor that can be released via exosomes and exposed at the cell surface by many tumor cells or, in response to stress, by DCs (39, 47). Another known ligand of NKp30, B7-H6, is expressed on transformed cells, and its expression can also be induced on normal cells (including monocytes and neutrophils) following stimulation with TLR ligands or pro-inflammatory cytokines (40, 48). Finally, an NKp44-ligand has been identified as an exon 21spe-containing isoform (21spe) of mixed lineage leukemia-5 (MLL5) protein. While the MLL5 is expressed in the nucleus of normal cells and is involved in the regulation of cell cycle and hematopoietic differentiation, the 21spe-MLL5 is located in the cytoplasm and at the cell surface, where it cannot conceivably exert its physiologic function (41). Remarkably, this isoform appears to be exclusively transcribed in tumor cells.

Although it is presently unknown whether the tumor-associated NCR-ligands could also be induced by viral infections, there are evidences that certain viruses, such as filovirus or HIV, can promote the expression of still undefined NKp46- or NKp30-ligands on DCs or T cells (49, 50) Moreover, CMV-infected DCs can activate NK cells via NKp46 and DNAM-1 (51). Intriguingly, the mAb recognizing 21spe-MLL5 had been previously shown to react with CD4^+^ T cells treated with a specific peptide of HIV gp41 protein or derived from HIV-infected patients (50). Whatever could be the pathogenic process that mainly shapes the specificity of the NCRs, it is quite evident that these receptors are nevertheless involved in tumor surveillance *in vivo*. Experiments on NKp46KO mice support this evidence (52). In addition, various human studies have shown that both solid and hematologic malignancies can be frequently associated to the presence of NCR^dim^ NK cells (either in PB or at the tumor site) (53–56). In this context, different tumor-orchestrated mechanisms capable of suppressing NCR functionality have been described. Hypoxia, which frequently characterizes tumor tissues (57), or various factors produced by tumor cells or induced on by-stander cells, can down-regulate NCR expression or function. These factors include: IDO, TGFβ, PGE_2_ (58–60), or inhibitory NCR-ligands, such as the soluble form of BAT3 (61), and the proliferating cell nuclear antigene (PCNA) (which has been recently shown to induce NKp44-mediated inhibitory signaling) (see also Table 1B).

Besides inducing natural cytotoxicity, the NCRs can also orchestrate regulatory functions. Indeed, since their first discovery, these receptors were known to induce cytokine release. In addition, in the early 2000s NK cells were shown to participate to regulatory interactions with DCs, pDCs, neutrophils, macrophages, and T cells, involving the engagement of various receptors including NKp30 and/or NKp46 (12, 58, 62–67). Finally, over the past 10–15 years, different NCR-expressing NK cell types, poorly cytolytic, and prominently devoted to regulatory functions, have been described. According to recent findings, these various NK cell types and the classical NK cells appear to be part of the larger family of innate lymphoid cells (ILCs), which show, as unifying trait, the expression of the transcriptional repressor Id2 during their development from a putative common hematopoietic precursor (68, 69). The different ILCs can be induced in selected tissues and are characterized by unique cytokine patterns and surface phenotypic profiles (including or not NK cell markers). Roughly, in both mice and humans, three groups of ILCs, ILC-group 1, 2, or 3, can be defined according to their ability to release Th1-, Th2-, or Th17/22-type cytokines respectively. NK or NK-like cells are comprised within group 1 and 3. The ILC-group 1 includes classical circulating CD56^dim^CD16^+^ NK cells, and CD56^bright^CD16^dim^ cells (which prominently populate lymph nodes). Both these cell types can produce large amounts of IFN-γ and TNF-α upon NCR engagement (6). The ILC-group 3 includes the CD56^+/−^NKp44^+^NKp46^+^ IL-22-producing NK-like cells (NCR^+^ILC3), which populate mucosal tissues. NCR^+^ILC3 are not cytotoxic but, by producing cytokines including IL-22, maintain epithelial-cell barrier function and contrast bacterial dissemination. The role of NCR on these cells is still poorly defined, however, a recent report by Glazer et al. (70) indicated that NKp44 engagement could both induce TNF-α release and synergize with other cytokines to induce IL-22 production.

Another poorly cytotoxic NK cell population endowed with regulatory functions is represented by CD56^bright^CD16^dim^NCR^+^ NK cells that populate the decidua in the first trimester of pregnancy. These cells produce defined pattern of cytokines, chemokines, and pro-angiogenic factors [favoring the appropriate placenta and fetus development (71)], and participate at the induction of tolerance at the maternal/fetal interface (72). The interaction with trophoblasts by the engagement of NCRs would activate their regulatory functions (73, 74). Thus, the definition of the above-described NCR^+^ NK cell types, along with their new functions, would suggest that NCRs could recognize ligands expressed on different cells, to fulfill multiple tasks. In this context, for each receptor, different portions of the molecule and/or different polymorphism may have been shaped or selected to ensure the putative pleiotropy of NCRs (75–77).

## HLA-Specific Receptors: KIRs

**Figure 1:**
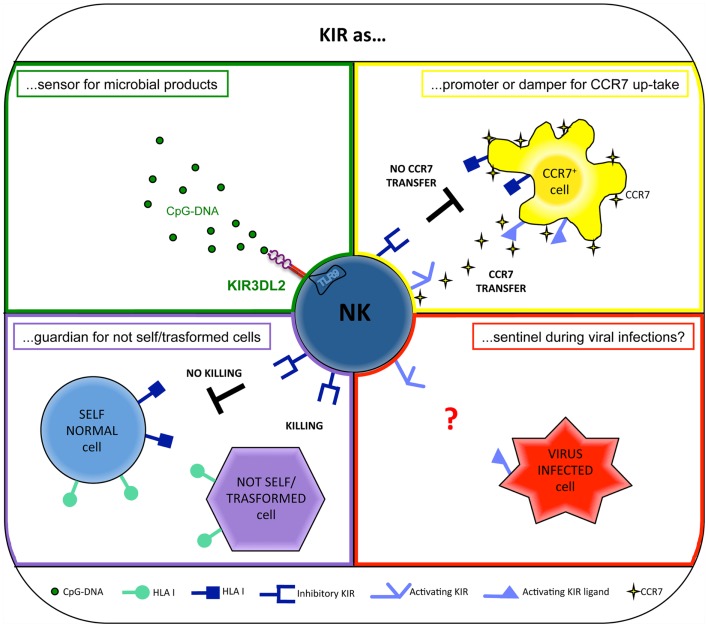
**Different roles for KIRs**.

Natural killer cells are equipped with inhibitory receptors able to interact specifically with human leukocyte antigen (HLA) class-I molecules on potential target cells. These receptors prevent NK cell-mediated attack against normal autologous cells and allow the killing of cells that upon tumor transformation or viral infection present compromised HLA class-I expression (“missing self hypothesis”) (78) (Figure 1). In humans, two different types of HLA class-I-specific inhibitory receptors exist: (i) killer Ig-like receptors (KIRs), also referred to as CD158, that belong to the Ig-superfamily and, in most instances, recognize the polymorphic HLA-A, -B, and -C molecules (79–81), and (ii) CD94/NKG2A (CD94/CD159a), a heterodimer related to C-type lectins that recognizes HLA-E (82), a non-classical MHC molecule characterized by a limited polymorphism.

In addition, activating forms of KIRs have been also identified, but their specificity is still largely elusive (see relating paragraph). Related to the function performed, the intracytoplasmatic domains of KIRs can feature either a short (activating KIRs) or a long (inhibitory KIRs) cytoplasmic tail, “S” or “L” in the nomenclature, respectively (79, 83) (Table 1C).

Each individual may differ strongly in the activating KIR content. In particular, two different types of KIR haplotypes (A and B) can be distinguished in humans. Both haplotypes share inhibitory KIRs, but haplotype A (in 50% of individuals) includes a single activating KIR (KIR2DS4) whereas haplotype B has up to five activating KIRs (83, 84). In general, haplotypes A are beneficial in NK responses to pathogens, whereas haplotypes B are associated with low frequencies of pregnancy disorders (85, 86).

In humans, 13 KIR genes and 2 KIR pseudogenes (KIR2DP1 and 3DP1) are expressed. Three conserved genes (KIR3DL3, 2DL4, and 3DL2) form the common framework.

Only some of the 13 human KIRs have been demonstrated to recognize HLA class I. In contrast, no ligand has yet been identified for KIR2DS2, 2DS3, 2DS5, 2DL5, 3DS1, and 3DL3 (87)(Table 1C).

Regarding the Ig-domains content, each KIR displays two (KIR2D) or three (KIR3D) extracellular Ig-domains. Two types of KIR2D can be determined. KIR2D of the first type are composed by D1 and D2 domains and include the majority of KIRs (KIR2DL1/L2/L3 and KIR2DS1/S2/S3/S4/S5), whereas KIR2D of the second type are composed by D0 and D2 domains and include KIR2DL4/L5.

The high level of polymorphism in the KIR gene complex and the low conservation of KIR genes between species (only the three KIR genes KIR2DL4, KIR2DS4, KIR2DL5 have been preserved through hominid evolution) have suggested that KIRs have been undergone to a rapid evolution (87). Following this process, new KIR alleles, generated from existing ones under evolutionary pressure, are likely maintained when providing advantages (88).

A clear diversity in the KIR gene complex between modern populations indicates that geographically distinct diseases have recently exerted a selection on KIR repertoires (89).

In addition, the KIR diversification is thought to be more rapid than HLA diversification. Indeed, HLA genes are more similar in humans and chimpanzees than their KIR counterparts. In humans, HLA-C seems to have evolved as a superior and more specialized ligand for KIRs as compared to HLA-A and -B. This fact is supported by two considerations: (i) all HLA-C allotypes are KIR ligands, whereas only 45% of HLA-A and 36% of HLA-B allotypes are recognized by KIRs and (ii) HLA-C is of more recent origin as compared to HLA-A and -B (90, 91).

## KIRs as Sensor for Microbial Products

Recently, KIRs have been shown to exert a novel and surprising function. Indeed, primarily KIR3DL2 (but also other KIRs, including KIR3DL1, KIR3DS1, and KIR2DL4) has been shown to work as sensors for microbial products and as chaperons for TLR9 ligands. In particular, KIR3DL2 can bind CpG ODNs at the NK cell surface and shuttle them to early endosomes where TLR9 translocates upon CpG ODN cell stimulation. In this intracellular compartment, KIR3DL2 gives CpG ODN to TLR9, thus allowing NK cell activation both in terms of increment of cytotoxicity and cytokine production (11, 19) (Figure 1).

The KIR Ig-domain involved in the direct recognition of microbial CpG ODN is represented by the D0, which is expressed by all the CpG ODN-binding KIRs. The interactions between negative charges of DNA sequences and positive charges of D0 domain probably are responsible for this binding. Remarkably, IFN-γ release induced by CpG ODN stimulation was mostly confined to KIR3DL2^+^ NK cell subset, suggesting that this receptor may be more efficient in CpG ODN-shuttling and NK cell triggering than the other CpG ODN-binding KIRs (19).

In this context, it is worth noting that KIR3DL2 represents a framework gene (92) and, as a consequence, NK cells of all individuals can bind CpG ODN. This novel functional capability of KIR3DL2 may provide an important clue to understanding the driving forces that led to conservation of KIR3DL2-encoding gene in all haplotypes, in spite of the low frequency of its HLA ligands (HLA-A3 or -A11 alleles) (93) in the human population. Indeed, the need of rapid NK-mediated anti-microbial responses may represent an important factor of selective pressure. Moreover, KIR3DL2 is characterized by poor inhibitory capability because of its low affinity for HLA-A ligands. This weak inhibitory function may explain why KIR3DL2, in most NK cells, is coexpressed with other inhibitory KIRs or NKG2A (19).

Remarkably, some studies have indicated a three-domain surface molecule carrying a D0 domain as the putative ancestral mammalian KIR and have suggested that all KIR2D encoding genes have evolved from a KIR3D-encoding gene. Considering these evidences, it is possible to hypothesize that, in origin, certain KIRs could have a role different from that to recognize MHC-class-I molecules (94).

## Activating KIRs as Sentinels during Viral Infections?

The main differences between activating and inhibitory HLA-specific receptors are located in their cytoplasmic tails. Indeed, the activating KIRs are characterized by a short cytoplasmic tail devoid of ITIMs and by a transmembrane domain with a charged amino-acid residue that allows association with an ITAM-bearing molecule (79, 95). The role of activating KIRs in immune response is still enigmatic and so far little information about it is available. Only for two of them (KIR2DS1 and KIR2DS4) the specificity for HLA class-I molecules has been demonstrated (96–100), despite their extracellular domains are characterized by a high degree of sequence homology with their inhibitory counterparts.

However, a number of experimental evidences suggest that activating KIRs may play a role in NK-mediated response against viral infections (Figure 1). In particular, protection against hepatitis C virus infection (101) and delayed progression to AIDS (102) have been described in individuals coexpressing the activating receptor KIR3DS1 and its putative ligand, HLA-Bw4 with an isoleucine at position 80 (HLA-Bw4-80I). In addition, high levels of degranulation by KIR3DS1^+^ NK cells in response to HIV-infected HLA-Bw4-80I^+^ CD4^+^ T cells (103) and a preferential expansion of KIR3DS1^+^ NK cells in HLA-Bw4-80I^+^ subjects during acute HIV-1 infection (104, 105) have been observed. However, the direct physical interaction between KIR3DS1 and HLA-Bw4-80I has not been demonstrated (106).

Moreover, different studies have suggested that the activating KIRs may interact with HLA class I at a lower affinity than their inhibitory counterparts. However, during viral infections, their HLA affinity may be increased by the presentation of viral peptides, thus allowing NK-mediated killing of infected cells (105). Thus, activating KIRs function could be modulated by the nature of the HLA class I presented peptide. In this context, KIR2DS1 has been described to display a certain degree of peptide selectivity in its binding to HLA-Cw4 (97).

It has been also speculated that additional molecules, up-regulated on target cells upon cellular stress/transformation, may also favor the triggering signals delivered by activating KIRs, acting as costimuli, or as non-HLA class-I ligands for these receptors. Notably, KIR2DS4 is able to interact with a non-HLA class-I protein expressed on melanoma cell lines and on a primary melanoma (107).

In some individuals, the increment in the signals generated by the low-affinity activating KIRs may overcome normal NK cell self-tolerance, thus inducing autoimmune diseases (108).

## KIR as a Promoter or Damper for a Phenotype Change

Since cellular function is often linked to phenotype, it is of particular interest that the recent discovery of a mechanism by which NK cells would be able to capture target cell membrane components, and incorporate them in its own membrane, thus enriching their phenotypic/functional features (109). This process, known as trogocytosis, involves several cell-surface molecules in different cell types including lymphocytes as well as DCs and tumor cells and may represent a vector for rapid intercellular communication (110). The capture of target cell membrane fragments by NK cells is likely to reflect specific ligand recognition by NK cell receptors and, in particular, activating and inhibitory receptors control this process. Importantly, surface molecules acquired from other cell types could modify not only phenotypic but also functional characteristics of the recipient cells (111).

Through this mechanism, some peculiar characteristics of the two major NK cell subsets (CD56^dim^/CD56^bright^) (10) can be subverted. In this context, recently, it has been shown that the highly cytotoxic CD56^dim^ KIR^+^ NK cell subpopulation that is characterized by the CCR7^−^ phenotype, can acquire surface CCR7 upon interaction with CCR7^+^ cells, becoming able to migrate in response to the SLC chemokines CCL19/CCL21 (7).

This novel NK cell ability occurs via the immunological synapse, precedes the NK-mediated cytolysis, and is finely controlled by the specific interaction between KIRs on NK cells and HLA class-I molecules on CCR7^+^ cells. In particular, inhibitory KIRs block this transfer (7), whereas activating KIRs (e.g., KIR2DS1) are able to strongly promote the CCR7 acquisition by NK cells (8, 20). In addition, other surface NK receptors, including the NCR NKp46, may play a crucial role in promoting this phenomenon (7). Therefore, in some individuals, collaboration between activating KIRs and other non-HLA-specific triggering receptors may occur to further potentiate the CCR7 acquisition.

Remarkably, unlike the non-HLA-specific receptors (whose function could be limited by inhibitory signals delivered by both KIRs/NKG2A receptors), KIR2DS1 could promote CCR7 uptake even in NKG2A^+^ NK cells, since the KIR2DS1-dependent uptake of CCR7 can override the inhibition provided by this receptor. This event allows a substantial expansion of the NK cell fraction capable of migrating to lymph nodes (20).

The key role of KIRs during the CCR7 transfer has important implications during haploidentical hematopoietic stem cell transplantation, in which donor-derived alloreactive NK cells expressing KIRs may play a relevant role in preventing graft vs. host disease (by killing recipient DCs) and host vs. graft reactions (by killing recipient T cells) (112). However, because KIR^+^ NK cells normally do not express CCR7, it was unclear how alloreactive KIR^+^ NK cells could reach lymph nodes and kill these cells directly in this compartment. In this context, the CCR7 acquisition by trogocytosis may represent a new way by which alloreactive KIR^+^ NK cells can migrate to the site where they can kill mature DCs and T cell blasts (20, 113) (Figure 1).

## Conclusion

Several data have demonstrated that the different NK cell receptors can reciprocally coordinate and regulate their functions, contributing to the initiation of innate responses and to the subsequent priming of adaptive immune responses. NK cell activation may be mediated by the engagement of TLRs and/or NCRs, two different types of receptors that may cooperate in inducing NK cell triggering, and thus in controlling viral/bacterial infection and cancer. For example, it has been demonstrated that TLR2 and NKp44 are directly involved in the recognition of *Mycobacterium* and in the consequent promotion of NK cell effector functions (28).

Natural killer cell triggering is under the control of receptors (e.g., KIRs) specific for self-HLA class-I molecules. The missing recognition of specific HLA ligands by inhibitory KIRs enables NK cell triggering upon NCRs engagement (37). Most tumor cell-surface ligands for NCRs have remained elusive; however, a critical role for NCRs in the control of both tumors and viral infections has been clearly demonstrated.

On the other hand, collaboration between TLRs and KIRs may also exist. Indeed, certain KIRs can function as sensor for microbial products and as chaperons for TLR9 ligands. Remarkably, an inhibitory KIR, such as KIR3DL2, may unexpectedly induce NK cell activation, favoring TLR-mediated response (19).

Activating and inhibitory NK receptors control other important mechanisms, such as trogocytosis, by which NK cells can modify their phenotypic/functional features. For example, KIR^+^ NK cells can acquire CCR7 and thus migratory properties toward SLCs, by interacting with CCR7^+^ cells (e.g., mature DCs). This process is blocked by inhibitory KIRs and promoted by NCRs and, even further, by activating KIRs (7, 20).

The NK-mediated capability of releasing chemokines and cytokines in different compartments endowed NK cells with regulatory functions, affecting subsequent adaptive immune responses. In decidual tissues, the engagement of NCRs during NK–trophoblast interactions induces the release of regulatory chemokines, involved in tissue building and remodeling, and in the formation of new blood vessels (74).

**Figure 2:**
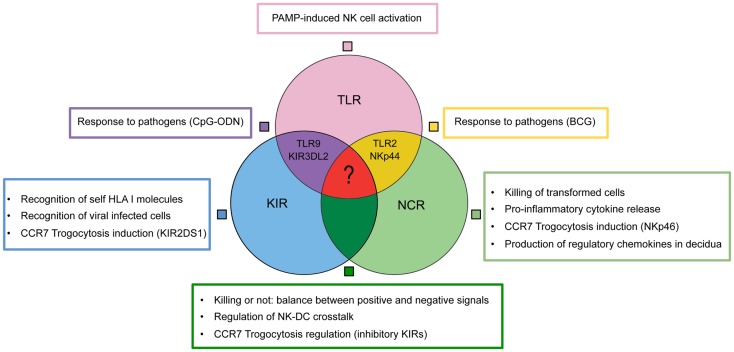
**Collaboration model between TLRs, KIRs, NCRs for NK cell activation**.

In conclusion, a wide and heterogenous group of receptors allow NK cell to fulfill multifaceted functions (Figure 2). The nodes of this intricate functional network may represent new therapeutic targets in different pathological conditions including not only tumors or infections but also immune-mediated diseases or pregnancy failure.

## Conflict of Interest Statement

Alessandro Moretta is a founder and shareholder of Innate-Pharma (Marseille, France). The remaining authors declare no competing financial interests.
